# Ending 2020 With a Special Theme Issue on Women's Services

**DOI:** 10.31486/toj.20.5016

**Published:** 2020

**Authors:** Ronald G. Amedee

**Affiliations:** Head of Clinical School and Professor, The University of Queensland Faculty of Medicine, Ochsner Clinical School, New Orleans, LA; Editor-in-Chief, *Ochsner Journal*

This year's holiday messages are reflective of the unprecedented times we’re in. Our messages to one another will focus on the fact that we made it to 2021 and we’re alive, and we’re ready for a fresh start.

–Megan Samarin

At the time that I’m writing this introduction, we are clearly amid the third surge of COVID-19 in the United States during 2020. The bright light at the end of this long and difficult journey is that vaccines are nearer to being approved than at any point previously this year. We are all ready for a fresh start and look forward to the possibility of a less tumultuous 2021.

The theme of this special issue is women's services. Highlights include 5 original research papers, including “Retrospective Analysis of Route Selection for Hysterectomy for Benign Indications at Ochsner Baptist Hospital” authored by Blosser, Morris, and Gala and “Gestational Age of Delivery in Pregnancies Complicated by Diabetes” by Harper, Tita, Biggio, and Chang. Lawson, Smith, and McCue offer a detailed look at the Ochsner Obstetrics and Gynecology Simulation Program, and we have 8 unique reviews and contemporary updates focused on topics as diverse as the prevention of preterm delivery, use of botulinum toxin A in the treatment of urogenital pelvic floor disorders, use of nitrous oxide for labor analgesia, prevention of surgical site infections in gynecologic surgery, use of technology to improve women's health care, and the association between genetics and gynecologic cancers. This edition also includes 4 case reports on women's health topics that truly round out this final edition of 2020.

The entire Publishing Services team at Ochsner Health is to be complimented for their tireless work throughout the pandemic in delivering both quality and pertinent manuscripts that have complemented our medical practice learning this year. On behalf of the *Ochsner Journal* Editorial Board and the editorial staff, I send you and your families our best wishes for the happiest of holidays, as well as a wish for a brighter and more positive New Year.

